# Hemorrhoids*Read at Palestine meeting, East Texas Medico-Chirurgical Association, in May, 1906.

**Published:** 1906-11

**Authors:** R. W. Skipper

**Affiliations:** Lovelady, Texas


For Texas Medical Journal.

### Hemorrhoids.*

BY R. W. SKIPPER, M. D., LOVELADY, TEXAS.

This is a subject that has been treated by great men, both in our text-books and journals, and it may appear to be presumption on my part to attempt to add to what has been said by them, but my excuse is that I have "something new under the sun."

What I have to say will not be on the pathology, etiology nor symptomatology of this loathsome and painful disease, but will have special reference to its treatment. The condition is too well understood by each of you to require any description here.

The lines of treatment laid down are many, from which the rectal surgeon may select that which he considers most suitable for the case in hand. Surgeons differ widely as to methods, some preferring

*Read at Palestine meeting, East Texas Medico-Chirurgical Association, in May, 1906.

the clamp and cautery, some the ligature and scissors, others puncture the hemorrhoid with needle that is properly charged with electricity, which causes it to slough or atrophy, while still others inject the pile with a fluid which causes sloughing and usually the disappearance of the morbid growth.

All of these proceedures are perfectly orthodox and safe except the last, which is fraught with dangers, yet it is practiced by some who are classed as surgeons.

The agent used by most of these is carbolic acid in some form, and many accidents have happened as a result of this practice.

This fact can be proven by an abundance of testimony, some of which will be adduced here, and some accidents named which follow the injection plan. Dr Joseph M. Mathews, of Louisville, Ky., in his work on "Diseases of the Rectum," speaks in strong terms against it, and substantiates his assertions by many quotations from good rectal surgeons; he says himself that death may occur from peritonitis, from embolism and from pyemia.

One of his quotations is from Dr. Andrews, who reports the following accidents which occurred in a series of 3304 cases: Deaths, 13; embolism of the liver, 8; sudden and dangerous prostrations, 1; abscess of the liver, 1; dangerous hemorrhage, 10; permanent impotence, 1; stricture of rectum, 2; violent pain, 83; carbolic acid poisoning, 1; failure to cure, 19; severe inflammation, 10; sloughing and other accidents, 35.

These statistics were gathered from the itinerants themselves, whom Dr. Mathews says will not report all their accidents.

In Sajous' Annual and Analytical Cyclopedia of Practical Medicine we have the following: "Piles should not be injected when inflamed, strangulated, large and hypertrophied, or external. When they are injected promiscuously, the treatment will frequently be followed by great pain, swelling, sloughing, abscess, fistula, phlebitis, pyemia, long delay from business, partial cure and occasionally death."

The same writer lays down fifteen rules which he says must be observed in order to avoid accidents, some of which it would be difficult to follow.

Hare, in his "Therapeutics," has this to say: "The injection of carbolic acid into hemorrhoids is a dangerous practice, and if employed only one drop is to be used." With this very laconic and forcible language he dismisses the subject.

If we had nothing else to convince us of the dangers spoken of here, this alone ought to be conclusive, coming as it does from Prof. H. A. Hare.

Gould and Pyle, in a recent work, "Cyclopaedia of Medicine and Surgery," says that the pile may be injected with a solution of carbolic acid consisting of equal quantities of acid, glycerin and water, and only four or five drops to be used, but that it must be repeated several times, requiring weeks to effect a cure.

Dr. Joseph D. Bryant speaks of the method only to condemn it.

In the *Therapeutic Gazette* for March of the current year, we have a symposium on the treatment of hemorrhoids, written by seven good surgeons, and each one condemns it in strong terms. I will refer you to those articles as being among the best and most recent on the subject.

After weighing all this testimony we are forced to the conclusion that the injection of pile tumors with carbolic acid of any strength is not safe, nor does it effect a cure in nearly all the cases so treated. Those of you who have read an article on "Carbolic Acid," written by me and published in the *Texas Medical News* of February, 1905, by order of the Houston County Medical Society, are able to anticipate what I shall have to say here in regard to the use of this excellent drug.

I have given my technique of its use in both hemorrhoids and carbuncle, and shall now again endeavor to explain its use in the former disease. I am sure that if you will adopt the method you will have very few cases that require a more elaborate treatment for their cure, or that will leave you and go off to the "advertiser" to have their piles injected, taking the risks that have been shown to attend that operation.

My plan is a perfectly safe one, does its work well, and can be applied to all cases when the tumors can be seen, consequently it does away with all other measures in most cases that come to us for treatment. The acid, when applied in its pure state, causes the parts to mummify or shrivel, and this is what it does when applied to pile tumors, curing them quickly, safely and pleasantly. It coagulates the fluids in the superficial tissues, and then there is no possibility of the poison going beyond the eschar produced by the burn. This burn is quite superficial, yet it is deep enough to destroy the film of mucous tissue overlying a hemorrhoid or to cause an external pile to atrophy.

The process is a little slow, yet in twenty-four to forty-eight hours the thrombotic pile will shed its clot and other varieties will be much better than when first treated, and usually in from one to two weeks your case will be entirely cured. I have been asked if I had relapses after my method of treating. I have learned of some who had a recurrence, but this should not condemn my method of treatment, for we have like relapses after other surgical measures have been resorted to.

With my plan we have no deaths, embolism, sudden or dangerous prostrations, no abscesses of the liver, nor dangerous hemorrhages, no impotence, no stricture of the rectum, no violent pain nor carbolic acid poisoning, no severe inflammations, no sloughing nor accidents can happen; we have no peritonitis nor pyemia, we have no phlebitis, no swelling, no long delay from business nor partly cures, but our motto is as stated above, Curare "Cito, Tuto et Jucunde." Tumors are never too large nor too much inflamed or strangulated. Both internal and external piles are successfully treated.

No preparatory treatment is necessary, except to empty the lower bowel by enema or by the administration of a purgative, followed by an enema. You may use a local anesthetic if you deem it necessary, which is only required in nervous individuals, for carbolic acid is itself a good local anesthetic, and usually abolishes nearly all pain.

Now for the technique. Have all tumors exposed in the usual way, and when they have been well cleansed, apply pure carbolic acid to every one, going slowly and carefully using only enough of the drug to make each pile white on its surface, and see that none diffuses itself beyond the field of operation. The tumors should be well dried by the use of absorbent cotton. You may use a camel's-hair pencil or a feather to apply the acid, shaking off any superabundance that may adhere to it so as to carry but a small quantity at a time, thus avoiding any burn. If there is much pain, either from the inflamed condition present or from the burn, you will certainly give your patient an anodyne. This is not often required for reasons above stated.

The after-treatment is simple. The parts are as nearly aseptic as you could make them, for the acid in its pure state destroys all bacteria, consequently it is only necessary to apply a simple dressing of vaseline or other agent that will keep the parts moist, and then cover with cotton which must be held in place with a T bandage.

The emolient may be used by the patient as often as he may desire, and the acid should be used as often as the case requires it, being used only by the surgeon. The parts must be cleansed as well as possible before each subsequent application; no two cases will require the same amount of care. Nearly all of my cases have been cured within a week, but some have required a longer time.

We should not be discouraged if we should have to treat some longer, for many cases do not get well under any line of treatment sooner than this. Since we know alcohol to be a perfect antidote to carbolic acid poisoning, we can use it after our applications are made if we fear an excoriation of parts beyond our field of operation. Do not use it, however, unless there are positive indications for it, for you will retard a cure if you do.

I wish now to relate the history of a few cases that I have treated and I will have finished:

Case 1 (1895): B. S. came to my office suffering severely with one large thrombotic pile protruding, strangulated, and being chafed by every movement made in walking. He being a farmer and being too busy to quit work, asked me if I could keep him on his feet, which I did not promise to do. I gave him the treatment as outlined above and told him to call on the following day, but did not see him again for several weeks. I then asked him why he had not returned, and was surprised to hear him say that he kept on plowing and was well in a few days, having but little trouble after having had the application made.

Case 2 (1898): J. O.; came with a number of large tumors, some of which were thrombotic. He also desired to be kept at his business, and I was more sanguine now, and told him I thought I could cure him without having him give up his work. Hartshorne says, "experience lends confidence," and it is true. He received his first treatment and went away to return only after three days, but said that he had been so much benefited that he did not deem it necessary to return sooner. He was then treated three days in succession and discharged, cured.

Case 3: Soon after this Mrs. L. came with one small but very painful tumor, and was not only relieved, but cured by one application.

Case 5: Mrs. H.; pregnant eight and one-half months, and with one thrombotic pile which was enormous in size, and several others not so large; had been given the whole line of palliatives, but to no avail. She sent for me because her family physician was sick, and asked me if I could relieve her. I told her I could. She had slept very little in ten days, so she told me, and was unable to stand on her feet. She declared in a short time after her first treatment that she had not been so easy in many days. After receiving five treatments in as many days, she was cured.

Case 6: Mrs. P: pregnant eight months; had one medium-sized tumor, highly inflamed and severely strangulated; was cured by one application. I delivered her at term and found no hemorrhoid.

Case 7: This case was peculiar, being of twelve years' standing, and presenting the greatest mass of hemorrhoids I ever saw. They had been strangulated on several occasions, and required the services of a physician to reduce them. They came down quite often, and, to use his own words, "he had to put them up at the end of every long row plowed, and often in the middle." I had to make a crucial examination, to satisfy myself that it was not a case of rectal prolapse. He had experienced severe hemorrhages and was a very forlorn patient, yet it was with much reluctance that he agreed to be treated. The multiple tumors were well cleansed and painted until white and then pushed back home, for he declared he could not leave them down. For two or three days he had considerable pain, and one of his bleeding spells, which was the last he ever had. The tumors then remained on the outside, the salient point of each being slightly ulcerated, and in a few days they were practically gone. Within two or three weeks he was well, except a tumor about the size of a split pea, and has had no further trouble during a period of sixteen months. The acid was used but one time and the subsequent dressing was that named elsewhere.

